# Time Free From Hospitalization in Children and Adolescents With Cystic Fibrosis: Findings From FEV_1_, Lung Clearance Index and Peak Work Rate

**DOI:** 10.3389/fped.2022.926248

**Published:** 2022-06-22

**Authors:** Simone Gambazza, Alessandra Mariani, Anna Brivio, Federica Carta, Chiara Blardone, Saba Lisiero, Maria Russo, Carla Colombo

**Affiliations:** ^1^Fondazione IRCCS Cà Granda Ospedale Maggiore Policlinico Milano, Healthcare Professions Department, Milan, Italy; ^2^Fondazione IRCCS Cà Granda Ospedale Maggiore Policlinico Milano, Cystic Fibrosis Centre of Milan, Milan, Italy; ^3^Department of Pathophysiology and Transplantation, University of Milan, Milan, Italy

**Keywords:** cystic fibrosis, lung clearance index (LCI), exercise tolerance, lung function, hospitalization, pulmonary exacerbation

## Abstract

**Background:**

An exercise test combined with a multiple breath washout nitrogen test (MBWN_2_) may offer a comprehensive clinical evaluation of cystic fibrosis (CF) disease in children with normal spirometry. The purpose of the present study is to explore whether information derived from spirometry, MBWN_2_, and exercise tests can help the CF multidisciplinary team to characterize time free from hospitalization due to pulmonary exacerbation (PE) in a cohort of pediatric patients with CF.

**Methods:**

This prospective observational study was carried out at the Lombardia Region Reference Center for Cystic Fibrosis in Milano, Italy. In 2015, we consecutively enrolled children and adolescents aged <18 years with spirometry, MBWN_2_, and Godfrey exercise test performed during an outpatient visit.

**Results:**

Over a median follow-up time of 2.2 years (interquartile range [IQR], 2.01; 3.18), 28 patients aged between 13.0 and 17.4 years were included. When lung functions were outside the normal range, 50% of patients were hospitalized 4 months after the outpatient visit, and their response to exercise was abnormal (100%). Half of the individuals with normal forced expiratory volume in the first second (FEV_1_) and abnormal lung clearance index (LCI) experienced the first hospital admission 9 months after the clinic visit, and 84.2% presented an abnormal response to exercise. Conversely, 15.8% had abnormal exercise responses when lung functions were considered normal, with half of the adolescents hospitalized at 11 months.

**Conclusion:**

Maintaining ventilation homogeneity, along with a normal ability to sustain intense work, may have a positive impact on the burden of CF disease, here conceived as time free from hospitalization due to PE.

## Background

Multidisciplinary care has achieved remarkable improvement in health outcomes for people with cystic fibrosis (CF), substantially changing the natural trajectory of the disease. Despite a stall in lung function decline from childhood into early adulthood (1), CF continues to limit the quality of life, particularly when hospitalization to treat pulmonary exacerbation (PE) is required (2–4).

Pulmonary exacerbation remains an important clinical event in the course of CF that increases the risk of lung transplant and mortality (5, 6). Predicting PE before the onset of any signs or symptoms remains challenging. Currently, bronchoalveolar lavage (BAL) and computed chest tomography (CT) are the gold standards to demonstrate airway inflammation during silent periods; in addition, these have been shown to predict PEs requiring hospitalization (7). However, neither BAL nor CT is frequently repeatable in a pediatric clinic. Yet, relying on tools to monitor early lung disease in a non-invasive and cheap way is warranted (8).

During the last 10 years, sensitive and comprehensive markers of lung disease have been explored (9). The nitrogen multiple breath washout (MBWN_2_) tests yielded one of the most promising results. The Lung Clearance Index (LCI) was shown to be more sensitive than spirometry to detect CF lung disease (6). It is repeatable and correlates with the results of high-resolution CT outcomes. Due to its attractive feasibility and clinimetric properties, it is particularly indicated for young children with CF and patients with early or mild CF lung disease (10). Another interesting field being progressively explored in CF is exercise capacity. The European CF Society (ECFS) recommends the cycle ergometer Godfrey protocol with ventilatory gas analysis as the preferred method of cardiopulmonary exercise testing (CPET), which allows for prognostication in CF (11). When gas exchange equipment is not available, the Godfrey protocol is among the second-best options recommended. Peak work capacity (Wpeak) elicited during continuous incremental cycle ergometry was shown to be a valid metric of cardiorespiratory fitness in children with CF (12) and predictive of survival (11). Furthermore, it is associated with nutritional status, airflow obstruction, and the presence of chronic *Pseudomonas aeruginosa* infection in adult patients (13). Altogether, exercise tests combined with respiratory function measures offer a comprehensive clinical evaluation of lung disease in children with CF.

The purpose of the present study is to explore whether information derived from spirometry, MBWN_2_, and exercise testing can help the CF multidisciplinary team to characterize time free from hospitalization due to PE in a cohort of pediatric patients with CF.

## Methods

This prospective observational study was carried out at the Lombardia Region Reference Center for Cystic Fibrosis in Milano, Italy. From January 2015 to February 2021, children and adolescents aged <18 years were consecutively recruited if the following conditions were met: a CF diagnosis based on a positive sweat test (chloride >60 mEq/L) and/or the presence of two disease-causing mutations, spirometry, and MBWN_2_ tests carried out together no more than 4 days apart from the exercise testing. We also included patients on any CF transmembrane conductance regulator (CFTR) modulators. Tests had to be performed under clinically stable conditions, defined as no changes in routine therapy for the 1 month preceding each test. Individuals with *Burkholderia ssp*. infections are not allowed to perform the MBWN_2_ test per center protocol, thus they were not included in the present study.

Follow-up was ended when individuals experienced the first hospitalization, otherwise, they were censored at the time of the end of data collection. We only considered hospitalizations due to the need for additional antibiotic treatment to be administered intravenously following a change in respiratory signs and symptoms, which denotes the occurrence of PE in our study.

The demographic and clinical data for each participant were extracted from the available electronic health records. Written, informed consent signed by parents or guardians of the child was obtained. The study was reviewed and approved by the local ethics committee Comitato Etico Milano Area B (456/2021).

### Lung Function

Spirometry was performed according to American Thoracic Society (ATS)/European Respiratory Society (ERS) guidelines (14) and always after the MBWN_2_ test. Forced expiratory volume in the first second (FEV_1_) was converted in the percentage of predicted values (ppFEV_1_) and *z*-score (15). Patients' lung function was considered in the normal range when FEV_1_ was above the −1.64 *z*-scores (lower limit of normal [LLN] at 5th percentile) and when LCI was below 7.91. (16). MBWN_2_ was performed using the Exhalyzer^®^ D and Spiroware software (version 3.3.1; Eco Medics AG, Switzerland) in compliance with the Standard Operating Procedures by Jensen et al. (17). Only results from three reproducible runs, defined as a variation of functional residual capacity and LCI values within 10% were considered. FEV_1_, LCI at 1/40th of the starting concentration, and indices of ventilation inhomogeneity in the conductive (Scond^*VT^) and acinar (Sacin^*VT^) airway regions were considered as respiratory study outcomes for the present study. An adequate environment with adequate distraction for younger children was assured during each test (18).

### Godfrey Protocol

An incremental cycle protocol to volitional fatigue was performed in compliance with the Godfrey protocol, (19) consisting of a protocol of fixed watt (W) increments every minute, depending on the height and FEV_1_ of the individual performing the test (10 W h < 120 cm or FEV_1_ <30%, 15 W h = 120–150 cm or 20 W h >150 cm). The warm-up consisted of 3 min of unloaded pedaling. Patients were given strong verbal encouragement to exercise as long as they could, and the test was terminated if the patient could not maintain a cadence above 60 rpm. All tests were performed using an electronically braked cycle ergometer (CosMed, Rome, Italy). Parents/guardians of children provided additional written informed consent to exercise testing. The main performance measurement recorded was peak work rate (Wpeak), which was also expressed as a percentage of normal values (ppWpeak). The following criteria were used to identify an abnormal exercise tolerance: peak work rate below 93% of normal values, heart rate (HR) at peak exercise ≥15 beats per minute below estimated peak HR, oxygen saturation (SpO_2_) decreased by more than 4% or dropped below 90% (19).

### Statistical Analysis

Descriptive statistics were used to summarize demographic and clinical features by the median and interquartile range (IQR). The relationship between variables was assessed by Spearman's correlation (rho); precision was reported using a 95% confidence interval (*CI*) based on 1,000 bootstrap replications. For further analysis, we also explored the correlation between FEV_1_ *z*-score, ppWpeak, and LCI at 1/20th of the starting concentration (LCI_5%_). The median follow-up was calculated using the reverse Kaplan–Meier method and time-to the first hospitalization was estimated using the Kaplan–Meier analysis. All statistical tests were performed using the open-source software R Core Team, version 4.0.3. (20), with the *confintr* package added.

## Results

Over a median follow-up time of 2.2 years (IQR 2.01; 3.18), 28 patients were included; 14 were between 13.0 and 17.4 years old. Sample characteristics are shown in Table 1. In total, 21 of the children and adolescents (75%) presented with an FEV_1_ *z*-score within the normal range, which corresponds to a median ppFEV_1_ of 90.9 (79.1; 99.2)%. The MBWN_2_ showed that ventilation inhomogeneity was impaired in 23/28 (82.1%) of the participants; median LCI was 9.7 (8.2; 13.6) and peripheral airways presented with higher inhomogeneity (Sacin^*VT^), 0.187 (0.099; 0.296) compared with conductive airways (Scond^*VT^), 0.078 (0.061; 0.107). When using LCI_5%_, 23/28 (82.1%) participants were above the upper limit of normal (ULN) of 5.73, and the median LCI_5%_ was equal to 6.5 (6.0; 8.6). Overall, 5/28 subjects (17.9%) showed a normal lung function profile in terms of both FEV_1_ *z*-score and LCI.

**Table 1:** Sample characteristics.

***N***	**28**
**Age, yrs**	**16.5 (13.0;17.4)**
**Sex (%)**	
** Females**	**11 (39.3)**
** Males**	**17 (60.7)**
**Mutation (%)**	
** F508del/F508del**	**7 (25.0)**
** F508del/other**	**14 (50.0)**
** Other/other**	**7 (25.0)**
**BMI, z-score**	**−0.7 (−1.0; −0.7)**
***Pseudomonas aeruginosa*** **chronic infection (%)**	**15 (53.6)**
**Pancreatic insufficiency (%)**	**25 (89.3)**
**CFRD (%)**	**1 (3.6)**

*Data are expressed as median (IQR) or count (percentage). BMI = Body Mass Index; CFRD = Cystic Fibrosis Related Diabetes*.

**Figure 1:**
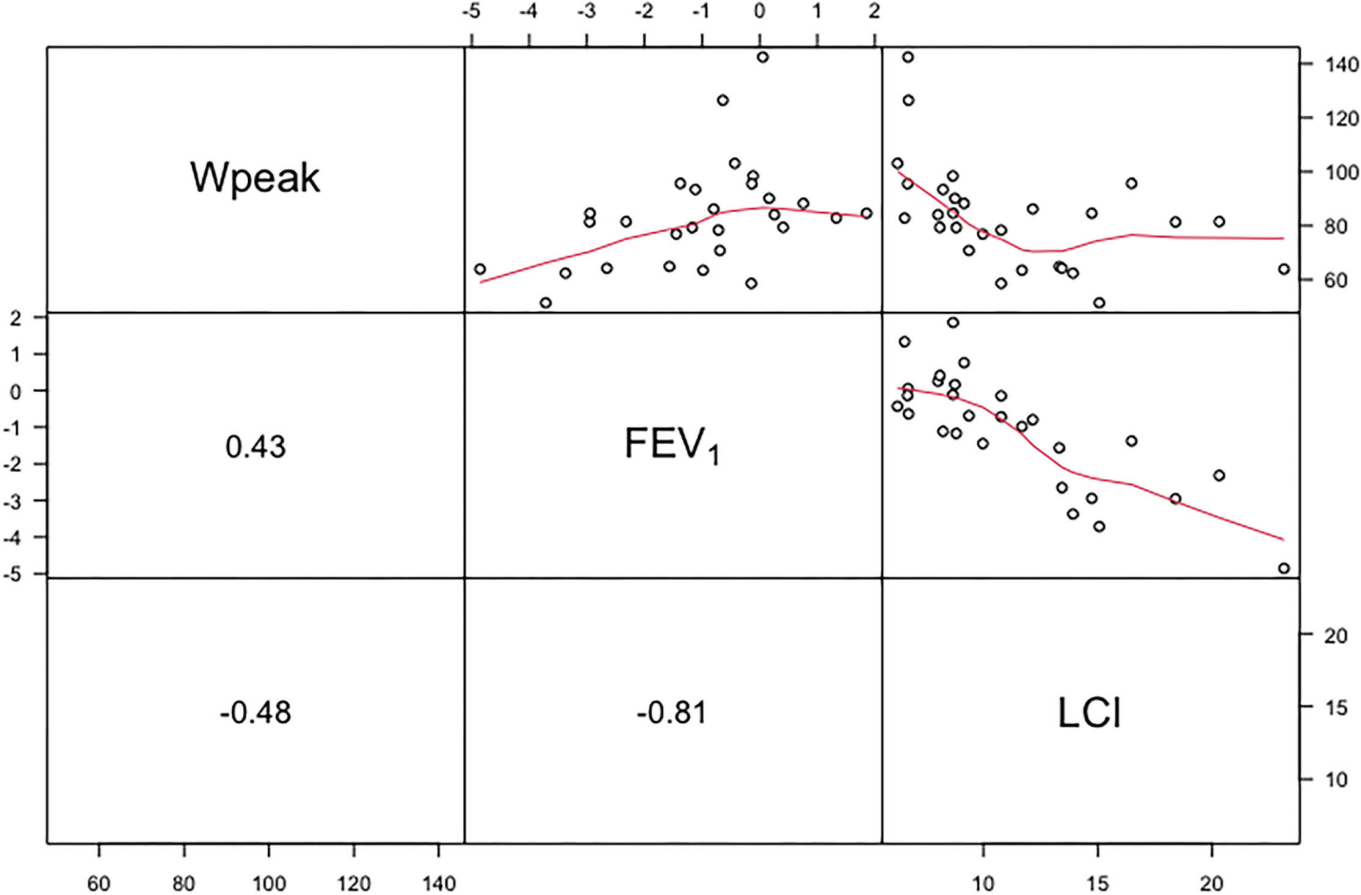
Upper panel shows the relationship between ppWpeak (Wpeak), *z*-score of FEV_1_ and LCI. The curved lines represent nonparametric regressions. The lower panel shows correlation coefficients (rho). FEV_1_, forced expiratory volume in 1 s; LCI, lung clearance index at 1/40th of starting concentration.

Figure 1 shows the relationship between ppWpeak derived from the Godfrey protocol with lung functions. A moderate positive correlation was found between ppWpeak and FEV_1_ *z*-score (0.43, 95% *CI*: 0.22; 0.73), whereas a negative correlation between ppWpeak and LCI was found (−0.48, 95% *CI*: −0.80; −0.23). The FEV_1_ *z*-score and LCI showed the strongest relationship (−0.81, 95% *CI*: −0.91; −0.61) among selected variables. The shape and strength of correlation with FEV1 *z*-score (−0.77, 95% *CI*: −0.87 to −0.53) and ppWpeak (−0.41, 95% *CI*: −0.74 to −0.10) were almost the same when adopting LCI_5%_.

During exercise testing, children and adolescents generated 140.0 (105.0–80.0) W, corresponding to 82.1 (69.3–90.9)% W predicted, and 92.9% (26/28) yielded an abnormal exercise response: 21/28 (75%) had abnormal Watt response, 20/28 (71.4%) had abnormal heart rate response and 1/28 (7.1%) experienced a drop in SpO_2_. Globally, 2/28 individuals presented a normal response to exercise and lung function within normal ranges. A summary of selected outcomes derived by exercise testing and MBWN_2_ of children and adolescents followed up in the present study are presented in Table 2, stratified by FEV_1_ LLN; 22/28 (78.6%) experienced one PE requiring hospitalization, and time-to-first hospital admission was 275 (IQR 125.0; 505.0) days.

**Table 2:** Outcomes stratified by FEV_1_ LLN.

	**FEV_**1**_ < LLN**	**FEV**_**1**_ **≥LLN**	
		**LCI < ULN**	**LCI ≥ULN**
*n* (%)	7 (100)	5 (23.8)	16 (76.2)
Age, years	17.4 (16.8; 17.6)	16.5 (14.8; 17.4)	15.8 (12.5;17.0)
LCI	15.1 (14.3; 19.4)	6.7 (6.6; 6.7)	9.3 (8.7;11.0)
Sacin*^VT^	0.240 (0.206; 0.440)	0.076 (0.069; 0.099)	0.194 (0.157; 0.296)
Scond*^VT^	0.098 (0.091; 0.115)	0.046 (0.030; 0.060)	0.078 (0.062; 0.108)
ppFEV_1_	64.6 (55.6; 66.5)	98.4 (94.9; 100.6)	91.6 (86.7; 102.1)
Watt, %predicted	64.2 (63.1; 81.4)	103.1 (95.5; 126.4)	81.7 (75.3; 88.7)
W_peak_ < 93%	7 (100)	1 (7.1)	13 (92.9)
Abnormal exercise response, %	7 (100)	3 (15.8)	16 (84.2)
Individuals with hospitalization, %	7 (100)	4 (19)	16 (76.2)
Time to hospitalization, days	125.0 (76.0; 223.0)	363.0 (280.0; –)	275.0 (134.0; 505.0)

*Data are expressed as median (IQR) or count (percentage). LCI = Lung Clearance Index*.

**Figure 2:**
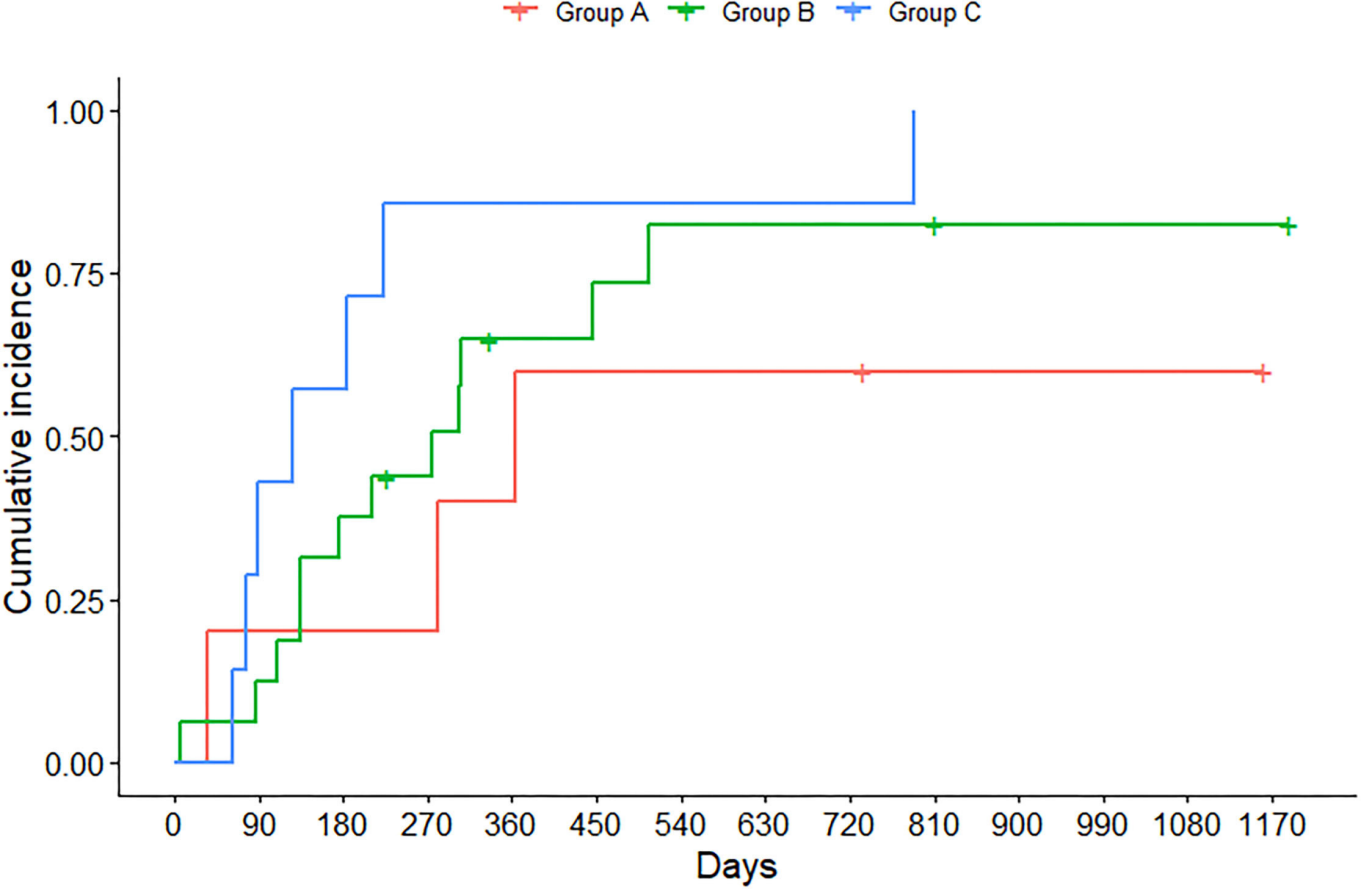
Time at which children and adolescents experienced hospitalization due to pulmonary exacerbation (PE), stratified by FEV_1_ *z*-score and LCI at 1/40th of starting concentration. Group A identifies individuals with FEV_1_ ≥ LLN and LCI < ULN; group B individuals with FEV_1_ ≥ LLN and LCI ≥ ULN, whereas group C individuals with FEV_1_ < LLN and LCI ≥ ULN.

Figure 2 shows that 50% of children and adolescents with lung functions outside the normal range (i.e., group C) experienced a hospitalization 4 months after the outpatient visit, and all presented an abnormal response to exercise as well. Half of the individuals with abnormal LCI and normal FEV_1_ (i.e., group B) experienced the first hospital admission 9 months after the clinic visit, yet presented an abnormal response to exercise, mostly due to the inability to generate a sufficient workload. Individuals in group A experienced PEs as well, but half of them had their first hospitalization at 11 months.

Further characteristics of treatment regimen of children and adolescents as identified by groups A, B, and C are summarized in Table 3. None of the children were on oxygen therapy; only one child was on continuous intravenous therapy due to *Mycobacterium abscessus* infection in the FEV_1_ < LLN group. Despite FEV_1_ being in the normal range, children and adolescents with LCI ≥ ULN showed the greatest therapeutic burden, mostly characterized by rhDnase and chronic treatment with azithromycin, due to the presence of *Pseudomonas aeruginosa*.

**Table 3:** Clinical characteristics and medical therapy details stratified by FEV_1_ LLN.

	**FEV_**1**_ < LLN**	**FEV**_**1**_ **≥LLN**	
		**LCI < ULN**	**LCI≥ULN**
*n* (%)	7 (100)	5 (23.8)	16 (76.2)
Age, years	17.4 (16.8; 17.6)	16.5 (14.8; 17.4)	15.8 (12.5; 17.0)
Severe genotype	5 (71.4)	4 (19.0)	13 (61.9)
*Pseudomonas aeruginosa* chronic infection (%)	4 (57.1)	2 (9.5)	9 (42.9)
Pancreatic insufficiency (%)	6 (85.7)	3 (14.3)	16 (76.2)
Lumacaftor/Ivacaftor	1 (14.3)	2 (40)	1 (6)
Elezacaftor/Tezacaftor/Ivacaftor	1 (14.3)	-	-
Antibiotics *per os*			
none	-	2 (9.5)	6 (28.6)
14 days	2 (28.6)	1 (4.8)	3 (14.3)
28 days	4 (57.1)	2 (9.5)	4 (19.0)
42 days	-	-	3 (14.3)
Chronic Azithromycin	5 (71.4)	2 (9.5)	11 (52.4)
Chronic inhaled antibiotics	5 (71.4)	1 (4.8)	8 (38.1)
Eradicating inhaled antibiotics	1 (14.3)	1 (4.8)	2 (9.5)
Other inhaled therapies			
rhDnase	4 (57.1)	5 (23.8)	16 (76.2)
Hypertonic saline 3%	-	1 (4.8)	-
Hypertonic saline 7%	4 (57.1)	-	6 (28.6)

*Data are expressed as median (IQR) or count (percentage). FEV_1_ ≥ LLN and LCI < ULN denote group A; FEV_1_ ≥ LLN and LCI≥ULN denote group B, and FEV_1_ < LLN denote group C*.

## Discussion

This study was conducted to explore time free from hospitalization due to PE, after a regular outpatient visit in a large CF clinic with almost 250 patients aged 6–18 years. In addition, the study aimed to investigate if the combination of spirometry, MBWN_2_, and exercise testing *via* the Godfrey protocol could characterize children and adolescents with CF. Our findings support the usefulness of a comprehensive approach based on lung function and exercise tests to identify children and adolescents with CF with the greatest burden of disease.

In the course of CF disease, PE is a meaningful event that has negative consequences on clinical outcomes (5, 21). Each PE occurrence poses a risk of permanent lung function decline, (22) and the frequency of PE is closely associated with a subsequent reduction in lung function either (21, 23). It is reported that ~25% of patients did not recover to the FEV_1_ baseline at the end of treatment (23). Most importantly, PE has significant effects on multiple domains of quality of life, which can take several weeks to return to the way they were before (3). In our cohort, 78.6% experienced one hospitalization due to PE, which occurred for the 50% of individuals between 4 and 16 months after a regular follow-up visit. In particular, half of the children and adolescents with both FEV_1_ and LCI outside the normal range (i.e., group C) experienced the first PE 4 months after the outpatient visit, and all presented an abnormal response to exercise as well. On the contrary, 50% of individuals with normal FEV_1_ and abnormal LCI (i.e., group B) experienced the first PE 9 months after the clinic visit, and the majority still presented an abnormal response to exercise, mostly due to the inability to generate a sufficient workload. The resulting 5-months-time free from hospitalization seems well defined by a substantial difference in MBWN_2_ indices and usage of antibiotics in the period preceding hospitalization. When both LCI and FEV_1_ are considered normal (i.e., group A), time free from hospitalization was remarkably high, and half of the adolescents experienced the event at 11 months. It is known that quality of life is associated with physical fitness, especially aerobic fitness (24), but exercise capacity is also affected by the number of antibiotic treatments and hospitalization (2). For instance, exercise capacity is way more negatively affected by PE managed at the hospital than at home (3). We might speculate that adolescents in group A had more time free from therapies, less severe CF disease, and therefore more time to get involved in physical activity, thus achieving better results at exercise testing compared with peers with greater therapeutic burden. This is also the group where there is the highest percentage of Lumacaftor/Ivacaftor use. However, previous studies have shown that Lumacaftor/Ivacaftor promotes gains in body weight and changes in body composition, reduces the rate of PEs (25) but does not affect exercise capacity (26).

Our results confirm the clinical utility of MBWN_2_ as a fundamental means to monitor lung disease in children with CF, tracking pulmonary abnormalities when spirometry is within the normal range (6, 27–29). Combining results from MBWN_2_ and exercise testing, we have further shown that LCI (both at 1/40th and 1/20th of the starting concentration) and ppWpeak are correlated, even in children with CF, and that these two measures can help profiling adolescents with normal FEV_1_. The bottom line is that CF teams should not assume that children with normal FEV_1_ are of good exercise tolerance, as already reported in adults (13). Furthermore, in our series 84.2% of children with normal FEV_1_ show abnormal exercise response when LCI is abnormal, compared to only 15.8% with normal LCI. It is likely that abnormal ventilation distribution, as assessed by LCI, could also be associated with less efficient ventilation during strenuous exercise, thus contributing to exercise limitations in CF lung disease, as previously demonstrated (30). However, the Godfrey cycle ergometer protocol without ventilatory gas analysis does not allow for direct measurement of ventilation efficiency and the primary measure is peak work capacity (Wpeak), which translates to the resistance level when exercise is terminated. Wpeak can be influenced by the type of protocol and tool used to run the exercise testing (31), but we use it as a surrogate for VO_2_ peak, because it can be measured extensively in the majority of Italian children with CF.

The combination of MBWN_2_ and exercise testing, even without gas analysis, is useful to identify children and adolescents with normal FEV_1_ with different ventilation inhomogeneity and exercise resistance. Such differences translate into 5 months of benefit at least, during which children with CF are not hospitalized. The results of this observational study provide objective insights on the need for advanced assessment during regular follow-up of children and adolescents with CF, particularly in the new scenario that is developing after the introduction of CFTR modulators into the market.

### Strength and Limitations

The major limitation of this study was the difficulty to recruit pediatric patients who were able to adequately perform all the required tests, and who had all the functional evaluations performed at the same time. Therefore, some selection bias might have occurred. Despite being valuable, MBWN_2_ and exercise tests require a long time to be executed, with enough staff-to-patients ratio to provide a comprehensive evaluation during regular follow-up visits, which is usually not the case in Italian CF centers (32). On the contrary, one of the greatest strengths is the long follow-up, and the fact that almost all the participants did not assume the triple CFTR modulator therapy. Indirectly, given the poor discriminative value of FEV_1_ in our study, this reinforces the need of more sensitive outcomes to evaluate young people with CF in the future, considering that there will be more and more patients taking new generation of CFTR modulators.

## Conclusion

Spirometry alone may no longer be sensitive to detect or monitor disease progression in this new era of CF care, especially in the pediatric age. Maintaining ventilation homogeneity, along with a normal ability to sustain intense work, may have a positive impact on the burden of CF disease, here conceived as time free from hospitalization due to PE. It remains important to focus our attention on the ability to exercise since childhood, in view of the need for increasingly sensitive tools to monitor the clinical status of young patients with CF.

## Data Availability Statement

The raw data supporting the conclusions of this article will be made available by the corresponding author, without undue reservation.

## Ethics Statement

The studies involving human participants were reviewed and approved by Comitato Etico Milano Area B. Written informed consent to participate in this study was provided by the participants' legal guardian/next of kin.

## Author Contributions

AM: conceptualization, investigation, data curation, and writing the original draft. SG: conceptualization, methodology, formal analysis, and writing the original draft. AB: investigation, resources, and data curation. FC, CB, and SL: investigation and data curation. MR: supervision and writing, reviewing, and editing the manuscript. CC: resources, supervision, and writing, reviewing, and editing the manuscript. All the authors approved the final manuscript as submitted and agree to be accountable for all aspects of the work.

## Conflict of Interest

The authors declare that the research was conducted in the absence of any commercial or financial relationships that could be construed as a potential conflict of interest.

## Publisher's Note

All claims expressed in this article are solely those of the authors and do not necessarily represent those of their affiliated organizations, or those of the publisher, the editors and the reviewers. Any product that may be evaluated in this article, or claim that may be made by its manufacturer, is not guaranteed or endorsed by the publisher.
